# Influenza Viruses: Harnessing the Crucial Role of the M2 Ion-Channel and Neuraminidase toward Inhibitor Design

**DOI:** 10.3390/molecules26040880

**Published:** 2021-02-07

**Authors:** Sphamadla E. Mtambo, Daniel G. Amoako, Anou M. Somboro, Clement Agoni, Monsurat M. Lawal, Nelisiwe S. Gumede, Rene B. Khan, Hezekiel M. Kumalo

**Affiliations:** 1 Drug Research and Innovation Unit, Discipline of Medical Biochemistry, School of Laboratory Medicine and Medical Science, University of KwaZulu-Natal, Durban 4000, South Africa; sphamtambo@gmail.com (S.E.M.); anou.somboro@gmail.com (A.M.S.); clegoni@gmail.com (C.A.); lawalmonsurat635@gmail.com (M.M.L.); gumedenelly0@gmail.com (N.S.G.); myburgr@ukzn.ac.za (R.B.K.); 2 Centre for Respiratory Diseases and Meningitis, National Institute for Communicable Diseases, Johannesburg 2131, South Africa

**Keywords:** influenza virus, influenza, neuraminidase, M2 channel, antiviral drugs

## Abstract

As a member of the *Orthomyxoviridae* family of viruses, influenza viruses (IVs) are known causative agents of respiratory infection in vertebrates. They remain a major global threat responsible for the most virulent diseases and global pandemics in humans. The virulence of IVs and the consequential high morbidity and mortality of IV infections are primarily attributed to the high mutation rates in the IVs’ genome coupled with the numerous genomic segments, which give rise to antiviral resistant and vaccine evading strains. Current therapeutic options include vaccines and small molecule inhibitors, which therapeutically target various catalytic processes in IVs. However, the periodic emergence of new IV strains necessitates the continuous development of novel anti-influenza therapeutic options. The crux of this review highlights the recent studies on the biology of influenza viruses, focusing on the structure, function, and mechanism of action of the M2 channel and neuraminidase as therapeutic targets. We further provide an update on the development of new M2 channel and neuraminidase inhibitors as an alternative to existing anti-influenza therapy. We conclude by highlighting therapeutic strategies that could be explored further towards the design of novel anti-influenza inhibitors with the ability to inhibit resistant strains.

## 1. Introduction

Influenza is a major cause of high morbidity and mortality through seasonal flu and global pandemics [1,2]. Seasonal influenza has resulted in 9–45 million illnesses and 12,000–61,000 deaths annually since 2010 [2,3]. Vaccination and anti-influenza drugs are the main current strategies used to prevent and treat influenza infections [4,5,6,7]. Antigenic drift or shift of human influenza viruses can result in new, highly virulent influenza strains that arise unexpectedly to cause new epidemics or worldwide pandemics [8,9]. The influenza virus mutates rapidly, which renders efforts to control the spread of the virus by vaccination inadequate [10,11].

These evolutionary mechanisms of viruses lead to the development of a variety of hybrid influenza viruses with different characteristics when compared to the parental viruses [12,13]. These variations make it difficult to control human influenza outbreaks through vaccination alone, since humans will not have immunity to this new virus subtype, thus increasing the possibilities of seasonal and sporadic pandemics [12,13,14].

The great Spanish 1918 H1N1 influenza pandemic with genes of avian origin resulted in approximately 50 million deaths in two years [15,16]. During that period, there were no effective vaccines or anti-influenza drugs. Thus, seasonal updates of influenza vaccines are essential to countermeasure changes in circulating influenza viruses. Even though vaccination is the primary strategy for prevention, in some seasons, protection cannot be rapid enough [17]. As such, the development of effective and safe anti-viral agents forms a significant component in the balanced approach of managing seasonal influenza and is critical for responding to new outbreaks of seasonal and pandemic strains.

Two major classes of anti-viral agents are currently available for treatment or prevention of influenza infections: M2 channel inhibitors, and neuraminidase inhibitors [18,19]. M2 channel inhibitors are only effective against influenza A viruses and are also associated with severe side effects and the emergence of drug resistance [20,21,22]. Neuraminidase inhibitors are a newer class of anti-influenza agents and they are effective against both influenza A and B viruses. Contrary to M2 channel inhibitors, they are associated with little toxicity and less drug resistance [23,24].

Currently no drug has been discovered that is effective against all influenza virus strains. This review will focus on the recent studies on the biology of influenza viruses as well as the structure, function, and mechanism of action of both M2 channel and neuraminidase influenza viruses. We also address the progress made in developing new M2 channel and neuraminidase inhibitors to offer more insights into possible therapeutic options.

## 2. Influenza Viruses

The human influenza viruses A (IAV), B (IBV), and C (ICV) belong to the *Orthomyxoviridae* family and have many common biological properties [25]. IAVs and IBVs are of epidemiological interest since they circulate and cause severe disease and major seasonal epidemics in the human population. On the other hand, ICV is associated with mild illnesses [5,26].

IAV and IBV are stabbed with two major surface glycoproteins (antigens) that dominate the virus surface: hemagglutinin (HA), and neuraminidase (NA) [27]. Both HA and NA perform complementary functions in the life cycle of the influenza virus. HA is responsible for the attachment of the virus to the host cell surface that is being infected. In contrast, NA is involved in the release of a progeny virion from an infected cell [27,28,29]. Conversely, ICV has a single major surface glycoprotein, the hemagglutinin-esterase-fusion (HEF) protein, which combines functions of both HA and NA [30,31].

IAVs and IBVs are conventionally named according to their species (if non-human), the location where isolated, the isolate number, a year of isolation, and lastly, the HA and NA virus subtypes in brackets. For example, A/Wisconsin/67/05(H3N2) was isolate number 67 of a human influenza A virus isolated in the state of Wisconsin in 2005, and it has an HA subtype 3 and an NA subtype 2 [32].

IAVs are classified based on the antigenic properties of HA and NA glycoproteins [33,34]. To date, 16 HA and 9 NA IAV subtypes, designated H1–H16 and N1–N9, have been discovered circulating in a wide range of aquatic birds [35,36]. These are expressed in several combinations of viruses isolated from aquatic avian species. An additional two combinations, H17N10 and H18N11, have been discovered in bats [37,38].

IBVs are instead divided into two antigenically distinct phylogenetic lineages, the B/Victoria/2/87 (B/Victoria) and B/Yamagata/16/88 (B/Yamagata) found circulating in seals [32,39]. ICVs have been isolated from humans and pigs. IAVs are more varied than IBVs, which are fundamentally exclusive to humans due to their capability to adapt to several species. IBV epidemics happen on average three weeks later than IAV epidemics [40,41,42].

New IAV and IBV strains arise regularly in a process referred to as antigenic variation (antigenic drift and antigenic shift) of HA and NA antigens [8,9]. This process inhibits the binding of neutralizing antibodies against common circulating strains, thereby allowing a new subtype of viral strains to avoid host immune response acquired through vaccination. These variations cause yearly outbreaks of influenza in the human population [43,44].

Antigenic drift is caused by intense selection pressure by the neutralizing antibodies of host immune systems, resulting in point mutations in the genes encoding NA and HA antigens. This drift leads to amino acid sequence changes in the antibody binding sites on these viral proteins. It occurs in both IAVs and IBVs [10]. The antigenic shift is due to the re-assortment of virus genomic segments when a cell is infected by two different strains of influenza viruses of different subtypes. It occurs only in IAV. This shift contributes to the replacement of genes encoding one or both surface antigens during replication, resulting in genome exchange [14,44].

### 2.1. Structure of Influenza Viruses

**Figure 1:**
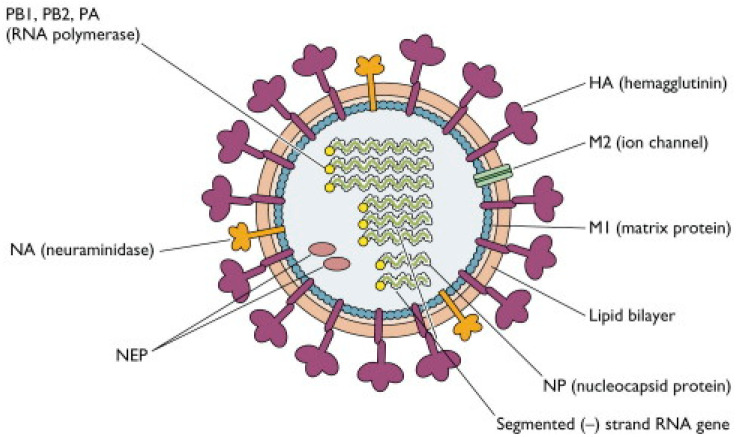
Structure of influenza A virus showing the two major surface glycoproteins (hemagglutinin (HA) and neuraminidase (NA)), the nucleocapsid and polymerase proteins (NP, PB1, PB2, and PA), the matrix proteins (M1 and M2), the non-structural proteins (nuclear export protein (NEP)), lipid bilayer and segmented negative-strand RNA genes [48].

By electron microscopy, IAVs and IBVs are both pleomorphic (spheres or very long filaments), with an average size of 100 nm in diameter for spheres and 300 nm in length for filaments. HA and NA glycoproteins project from the membrane surface as spikes. The two spikes differ in morphology—HA is triangular rod-shaped, while NA is mushroom-shaped (Figure 1). Each virion has an average of 500 HA and 100 NA spikes [45,46,47].

IAVs and IBVs contain eight negative-sense, single-stranded RNA genome segments and are encapsidated by nucleocapsid proteins to form ribonucleoprotein (RNP) [29,49]. They encode transcripts for 10 essential virus proteins categorized into four groups: (1) the nucleocapsid and polymerase proteins—nucleocapsid protein (NP), polymerase B1 protein (PB1), polymerase B2 protein (PB2), and polymerase A protein (PA); (2) the envelope proteins—HA and NA; (3) the non-glycosylated matrix proteins—matrix protein 1 (M1) and matrix protein 2 (M2) (NB and BM2 for IBV); and (4) the non-structural proteins—non-structural protein 1 (NS1) and nuclear export protein (NEP) [50,51,52,53,54,55].

In contrast, ICV only consists of seven RNA genome segments, and a single major surface glycoprotein, the HEF protein [30,31]. The lipid core interior of the virus particle is enclosed by the matrix protein (M1), covering the three integral membrane proteins: HA, NA, and CM2. A virus particle must contain each of all unique RNA segments to be fully infectious [56,57].

### 2.2. Replication Cycle of Influenza Virus

Human influenza viruses infect cells of the upper respiratory epithelium [58]. They are assumed to be transmitted from an infected person predominantly by aerosol or droplet infection, which is created while talking, coughing, or sneezing, thus contaminating the mucosa of the respiratory tract. Transmission can also occur via direct contact with virus-contaminated surfaces and successive mouth–nose contact. Following infection, the viruses replicate in the nasal and laryngeal mucosa [5,59].

**Figure 2:**
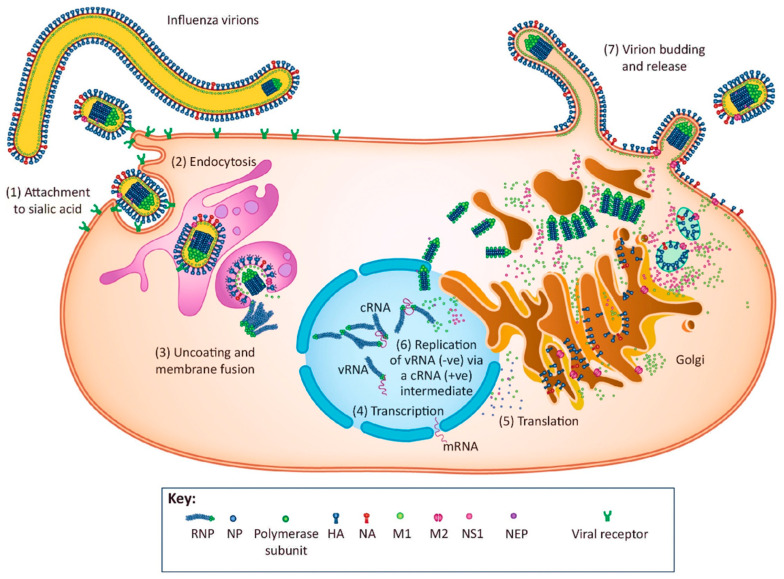
The replication cycle of influenza virus, illustrating seven discernible phases: (1) attachment; (2) endocytosis; (3) uncoating and membrane fusion; (4) transcription of the viral RNA; (5) translation of viral proteins; (6) replication of the viral RNA; and (7) virion budding and release [60].

Influenza virus replication can be divided into seven distinct phases (Figure 2): (1) virus attachment; (2) endocytosis; (3) uncoating and membrane fusion; (4) transcription of the viral RNA; (5) translation of viral proteins; (6) replication of the viral RNA; (7) virion budding and release.

#### 2.2.1. Virus Attachment

This is the first step in viral replication, where the virus binds to the host cell. Viral HA binds the influenza virus to the sialic acid of the cell surface glycoprotein or glycolipid that is being infected [26,61]. The sialic acid termini have two unique steric configurations, the α-2,3- and α-2,6- linkages. The HA proteins of human viruses prefer to bind sialic acid linked to galactose via an α-2,6-linkage (Siaα2,6Gal), which is predominantly found in human respiratory epithelial cells [62,63]. In contrast, HA proteins of avian viruses preferentially recognize an α-2,3- linkage (Siaα2,3Gal) that is predominant on the epithelial cells of duck intestines [64,65,66].

#### 2.2.2. Endocytosis

Following HA protein (or HEF in ICV) virus attachment to sialic acid-containing receptors on the host cell, virus particles enter the cell by clathrin-mediated endocytosis via clathrin-coated pits—a process by which receptors on the cell surface mediate an inward budding of the plasma membrane. This leads to the formation of endocytic vesicles (endosomes) containing the absorbed substances [67,68,69].

#### 2.2.3. Uncoating and Membrane Fusion

The acidity of the endosomal membrane influences the uncoating of the influenza virus. Low pH (~5) of the late endosome triggers host cell protease (trypsin-like) to cleave HA into two subunits (fusion peptide), HA1 and HA2 [70,71,72].

After cleavage, the hydrophobic free N terminus of the HA2 subunit (exposed fusion peptide) inserts into the endosomal membrane of the host cell. Fusion leads to the incorporation of the virus envelope with the endosomal membrane; this result in the opening of a pore through which Influenza A viral ribonucleoproteins (vRNPs) are released into the cellular cytoplasm. Uncleaved HA of influenza viruses can attach to, but not enter, the host cell and is consequently not infectious [73,74].

Additionally, in IAVs, protons influx through the M2 ion channel from the late endosome into the virus particle [75,76]. This influx leads to acidification of the interior of the virus particle leading to disruption of M1-RNP complexes, consequently enabling the release of vRNPs into cellular cytoplasm [77,78]. The M2 ion channel is also believed to prevent the premature activation of HA after cleavage by equilibrating the acidic pH of the Golgi apparatus [79,80].

Following release from the virion, cytoplasmic vRNPs are trafficked into the host cell nucleus by cellular import factors (nuclear localization signals), importin-α (karyopherin-α) and importin-β (karyopherin-β). The M1 protein, on the other hand, following separation from the vRNP complexes, is separately trafficked into the nucleus, where all vRNA synthesis takes place [77,81,82,83].

#### 2.2.4. Transcription of the Viral RNA

Inside the nucleus, viral RNA transcription is carried out by the viral RNA-dependent RNA polymerase (RdRp) complex (PB1, PB2, and PA subunits), whereby it binds to and cleaves the vRNA and concurrently leads to elongation [84,85]. The RdRp complex primes the vRNA template via a mechanism called “cap snatching” to increase initiation efficiency [86].

For cap snatching, the PB2 subunit binds to the 5′caps of the host mRNA, while the endonuclease activity of the PA subunit “snatches” (cleaves) 10–13 nucleotides downstream of the 5′cap. The produced 10–13 nucleotides with the cap serve as a primer for viral mRNA synthesis [54,87,88]. Synthesis is carried out by the polymerase activity of the PB1 subunit. Transcription finalizes by polyadenylation of the viral mRNA encoded by polyadenylation signal, an oligo-U sequence (5 to 7 Uracil residues) located close to the 5′-end of the template [87,88,89].

#### 2.2.5. Translation of Viral Proteins 

Synthesis of viral mRNA also occurs in the nucleus of the cell catalyzed by the same polymerase complex used for mRNA transcription, but without the requirement of a capped primer [90,91]. The viral polymerase uses the negative-sense vRNA as a template to synthesize a positive-sense copy of the vRNA termed complementary RNA (cRNA). The viral RNA polymerase subsequently transcribes this cRNA to produce more copies of vRNA [92,93].

The viral mRNA (vRNP segments) is exported from the cellular nucleus into the cytosol by the nuclear export proteins (M1 and NS2) for translation by the cytoplasmic ribosomes. The mRNAs transcribed by the RdRp complex are spliced by host cell machinery regulated by interferon-antagonist (NS1) protein to yield M and NS proteins. M1 is thought to form complexes with vRNPs, and NS2 mediates the export of the M1-RNP complex into the cytosol via nucleoporins [90,91].

#### 2.2.6. Replication of the Viral RNA

In the cytosol, the influenza virus protein synthesis is directly mediated by the host cell translation machinery. Viral RNA replication occurs in two steps: the first vRNA is replicated into cRNA, and then cRNA is copied into vRNA. Newly synthesized nucleoprotein (NP) and viral polymerase subunits (PA, PB1, and PB2) are imported back into the nucleus. These newly synthesized proteins are known to assist in viral mRNA transcription and vRNA replication [91]. Progeny vRNPs associate with nuclear export proteins and form a M1-RNP complex for trafficking towards the cell surface using microtubule-organizing centers (MTOCs) [94,95,96].

HA, NA and M2 membrane proteins are synthesized by ribosomes associated with the endoplasmic reticulum (ER). Following synthesis, they are trafficked into the Golgi apparatus for post-translational modifications (glycosylation of HA and NA, and palmitoylation of HA and M2) and subsequently directed to the cell membrane. HA, NA, and M2 integrate with vRNPs in the cell membrane and stick in the lipid bilayer for packaging [85,90]. The mechanism of packaging the eight vRNPs (seven for ICV) is currently not fully understood but is thought to be facilitated by segment-specific packaging signals. Influenza viruses with incomplete vRNPs are known to be not fully infectious [97,98].

#### 2.2.7. Virion Budding and Release

Influenza virus budding occurs in the lipid raft, a plasma membrane region known to be rich in sphingolipid and cholesterol [99,100]. The RNPs and M1 proteins aggregate in this membrane region, and when they reach high concentrations, they concentrate to create a virus particle. Budding is believed to be initiated by an accumulation of M1 matrix protein at the cytoplasmic side of the plasma membrane [20,101]. M2 has also been shown to accumulate at the boundaries of the budding sites and contribute to the scission of the virus particles [20,46].

The release of the newly assembled influenza virus bud is exceptionally dependent on the sialidase activity of NA to catalyze the cleavage of sialic acid from the host cell and virus glycoprotein [28,102]. As a result, HA is prohibited from binding to the cell surface, and the progeny viruses are released from the infected cell to spread the infection to uninfected cells [29,103].

## 3. The AM2 Ion Channel

Influenza A M2 (AM2) membrane protein is a type III integral membrane protein that is very selective for protons versus sodium and potassium ions. It forms a homotetrameric pH-regulated proton-selective channel located in the viral envelope [104,105,106,107]. Ion channel activity has been detected in *Xenopus oocytes* [106,107], mammalian cells [107,108], and yeast [109,110] expression systems. In the early stages of viral replication, the AM2 channel permits influx of protons from endosomes into the virus interior, leading to virus uncoating and the subsequent release of free RNPs into the host cell cytoplasm such that the viral genetic material can replicate [108,111].

The AM2 ion channel also plays a crucial role in the late stages of the viral replication cycle by working as a proton channel and equilibrating the pH of the Golgi apparatus with the cytoplasm. Thus, this prevents premature conformation change of the newly synthesized viral HA while they are transported to the plasma membrane of the infected cells [112,113]. The critical requirement for this viral protein makes it a good target for antiinfluenza drugs [114]. Evidence as to its functions has been mainly from studies of the action of drug-resistant mutants [110].

### 3.1. Structure and Function of the AM2 Ion Channel

The structure, mechanism of proton conductance, and inhibition of the AM2 ion channel were broadly studied by electrophysiology [22,115], site-directed mutagenesis [116,117], and molecular dynamics (MD) simulations [118,119]. However, the studies only established the overall topology and location of sidechains. Studies that employ high-resolution techniques such as solution NMR, crystallographic structures and solid-state NMR (SSNMR) have provided an elevated understanding of the proton channel to the atomic level [120,121,122,123].

**Figure 3:**
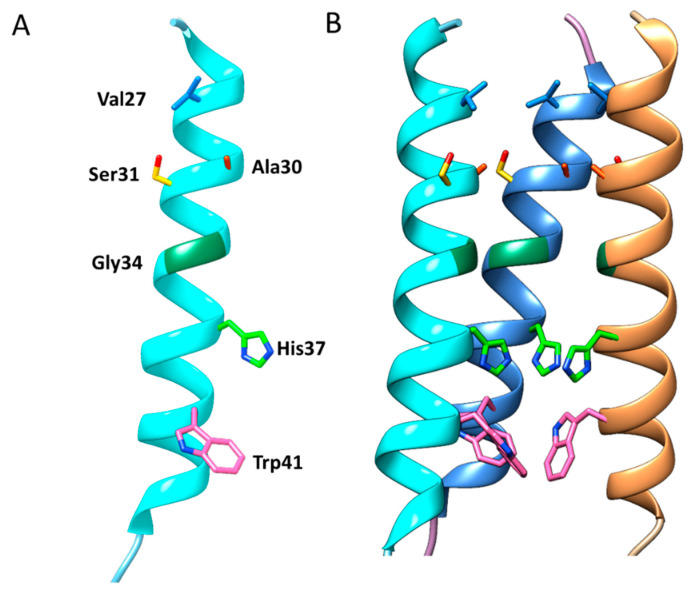
Three-dimensional structure of the influenza A M2 (AM2) ion channel. (**A**) A monomer of the AM2 protein transmembrane domain (TMD) displaying channel facing amino acid residues; (**B**) Organization of four TMDs, and the alignment of pore-lining residues. For clarity, three AM2 monomers are shown to expose the sidechains of the pore-lining residues. The NMR structure with PDB ID 2RLF was used (prepared by authors).

These studies determined that the three-dimensional structures of the AM2 ion channel (97 residues single-pass membrane) comprise three structural domains, which perform multiple functions (Figure 3). The *N*-terminal 23 residues ectodomain is responsible for the integration of AM2 into the virion [124,125]. Succeeding this region is a single transmembrane (TM) domain (19 residues), which is imperative for proton conductance, tetramerization of the protein, and drug binding [108,110]. Finally, the *C*-terminal cytoplasmic tail endodomain (54 residues) is critical for membrane localization, budding, scission, and binding to matrix protein M1, which is essential for the assembly and production of infectious virus particles [20].

The active site of the channel was established to be in the TM domain. The TM helices assemble into a four identical α-helix bundle with a left-handed twist angle of ~23° and a well-defined water-filled pore through which protons must pass to gain access to the viral interior [122,126,127]. Water molecules within the channel pore form a hydrogen-bonded water network known as the Grotthuss mechanism along the 17 Å stretch between the Val27 valve and His37 box.

The continuous highly structured network of water molecules is only observed in low pH conditions, compared to the intermediate pH 6.5 conditions, which shows less ordered waters [126,127]. Functional studies and crystallographic structures indicate that the ion channel pore is lined by Gly34, Ser31, Ala30, and Val27 sidechains with a tilt angle of ~25°.

The helices are firmly packed at the N terminus, and they are marginally spread-out toward the C terminus. At the *N*-terminal end, the ion channel pore entrance is narrowed to 2 Å by the hydrophobic sidechain of the Val27 valve and restricts water molecules from penetrating the channel [126]. The channel pore size progressively expands to an inner diameter of ~9 Å until Gly34. The channel then narrows at the half of the channel towards the C terminus, and the His37 and Trp41 sidechains form the narrowest points—too small to allow anything to pass. Trp41 obstructs the *C*-terminal end of the pore to a pore size of 1.4 Å in diameter [125,127].

His37 and Trp41 residues are located near the center of the TM domain. Four His37 sidechains are packed into a box-like structure (His-box) and individual imidazoles are connected by a structured network of water molecules via a low-barrier hydrogen bond (LBHB) [122]. The His-box needs to expand only slightly (1–2 Å) to permit the passage of water molecules [126]. The His37 sidechain acts as a proton sensor and conducts protons by protonation or deprotonation of its imidazole sidechain. The Trp41 sidechain forms a Trp-basket that acts as a pH-dependent gate of the channel [76,123,126].

This two-state gating mechanism has a structurally rigid closed state and loses the quaternary structure open state [128]. Cross-linking studies indicated that the four parallel TM helices are bound at one end of the N-terminus by intermolecular disulfide bridges at Cys17 and Cys19 [129,130]. Additionally, they are bound at the other end by *C*-terminal amphipathic (AP) helices (residues 51–59), ensuring that acid activation of the channel does not dissociate the tetramer [120].

In the closed conformation of the channel pore, the Val27 valve at the N-terminus and the Trp41 gate at the C-terminus effectively block water from freely diffusing into the pore from either side of the membrane. The four bulky Trp41 indole rings are at van der Waals (VDW) distance from each other, preventing the passage of water or ions [120,131]. Additionally, the Trp41 residue is suggested to form intermolecular hydrogen bonding with the carboxyl group of the adjacent Asp44 subunit to stabilize the closed Trp41 gate [126]. Mutating Asp44 to Asn triggered a significant increase in the activity of the AM2 channel, supposedly triggered by the disruption of Asp-Trp hydrogen bonding interaction [132,133].

Although IAV mutates and shuffles its genes, the coding regions for His37 and Trp41 residues are highly conserved in all known strains of avian, swine, equine, and human IAVs when compared with the other AM2 proteins encoded by the genome [134,135]. Mutagenesis studies have identified His37 and Trp41 residues as a function core of the channel. When His37 is replaced with either Gly, Ala, Glu, Lys, or Arg, the effectivity of the AM2 channel is reduced, indicating that His37 is essential for the proton selectivity of the channel [136,137]. Site-directed mutagenesis replacement of Trp41 with Ala, Cys, or Phe also results in the absence of the measurable pH-modulating activity of the channel at high pH, suggesting that Trp41 is the gate that blocks the fusion of protons from the inside but not from the outside of the virus [117].

A low pH medium destabilizes the TM helix–helix packing via electrostatic repulsion; this widens the pore to accept water molecules to enable His37 imidazole ring protonation [138]. This conformational change breaks the hydrogen bond between Trp41 and Asp44, enabling the Trp41 gate to flip open. The influx of protons goes through the channel into the virus interior to facilitate the separation of matrix protein and RNPs [117,123].

### 3.2. Catalytic Mechanism of the AM2 Ion Channel

The mechanism of AM2 ion channel activity has been thoroughly studied in oocytes, mammalian cells, and vesicles [106,107,108,109,110]. The interest in the ion channel stems from its proton selectivity since it has 10^6^ to 10^7^-fold more permeability to protons versus alkali metal ions such as sodium (Na^+^) and potassium (K^+^) under physiological conditions [111,121]. MD calculations, as well as functional studies, suggest that the channel responds solely to external pH. Low pH activates the channel and high pH closes the channel, irrespective of the interior pH. It conducts protons from the outside to the inside of the virus when the external pH is low, but does not as efficiently conduct protons outward when the pH gradient is reversed [139,140,141].

MD simulations and functional and spectroscopic studies of the AM2 proton transport mechanism have been extensively used to study the exact molecular mechanism of how protons are transported through the membrane, and they are still under debate. Two proton transport mechanisms have been proposed: an early model “water wire model” and the currently accepted model “proton relay model” [118,142,143,144,145].

According to the water wire model, protonation and deprotonation of His37 imidazole sidechains cause an electrostatic repulsion between charged histidine residues. This pushes tightly packed transmembrane (TM) helices apart, thereby opening the “tryptophan” gate and exposing His37 to proton acceptors (water). This repulsion results in the formation of a continuous water wire that shuttles protons from one water molecule to another [76].

According to the proton relay model, the His37 imidazole sidechain serves as a “relay” molecule, binding protons from the outside of the channel and releasing them to the inside of the channel by dissociation. This mechanism is assisted by tautomerization or flipping of the imidazole ring [110,122].

**Figure 4:**
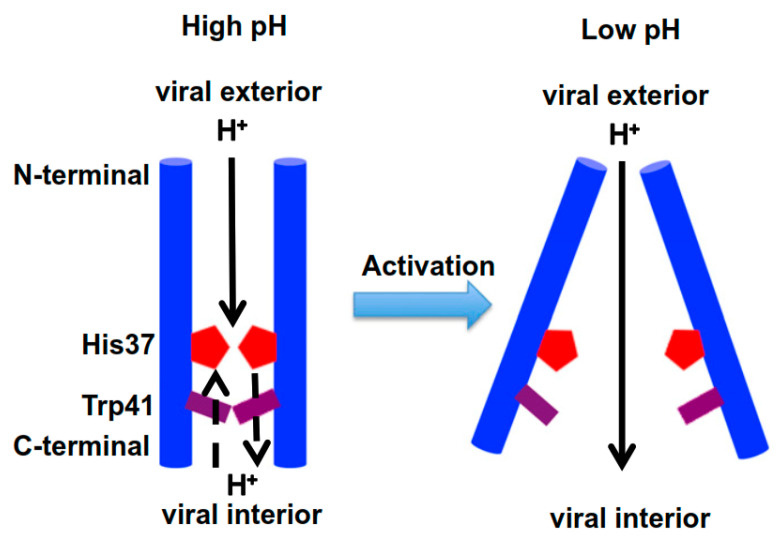
Model for AM2 channel acid activation and proton conductance displaying conformation change from closed to open conformers [148]. For clarity, only two helices and one protonation state are shown.

It has been suggested that the channel is closed when the pH_out_ exceeds pH 7.5 and is opened when the pH_out_ is lower than pH 6.5. Proton exchange is at the highest level between pH 5 and 6 of the endosome, where the +2 and +3 protonation states dominate [146,147]. MD simulations suggest that conformation change between C_closed_ and C_open_ conformers is stimulated by pH_out_ and typically takes place at the +3-protonation state of His37 imidazole rings (Figure 4) [127,141].

The relay model was further supported by solid-state NMR studies, which reported the first two protonation conduction steps of His37 residue occurring with pKa of 8.2, the third protonation at pKa of 6.3 and the fourth at pKa of ≤5 [147]. A different study reported the first two protonation steps of His37 tetrad to occur at pKa values of 7.6 and 6.8 [149]. These findings identified the shuttling of the third proton (conducting pKa) to occur near the midpoint of the conductance curve, suggesting that conduction transpires via the alternation of +2 and +3 states. Furthermore, MD simulations for possible protonation states were in agreement with the above studies [135,138].

Under a low protonation state of His37 (pH > 7.5), the Trp41 basket constricts the *C*-terminal pore below His37, forming a gate that blocks the influx of protons through the channel and dehydrates the His-box (the channel favors C_closed_ conformers) [76,138,149]. The NMR study indicated that lowering the pH from 7.5 to 6.0 caused immense broadening of most of the NMR resonances corresponding to the TM domain. The expansion was due to increased exchange between multiple TM domain conformations as the Trp gate opens and closes the channel [120,133].

A high His37 protonation state (pH < 6.5) favors the C_open_ conformers. As the pH decreases, the Trp-basket opens to expose protonated His37 molecules to the viral interior, able to enter primary proton conduction step, while the Val27 valve N terminal end of the bundle contracts [122,135,138]. When the highest protonation state is reached, the positive charge on the His-box increases and the Trp-basket opens sufficiently due to electrostatic repulsion between the His-tetrad. The open Trp-basket hydrates the His-box to create an aqueous conduction path, allowing the release of protons into the viral interior [123,135,138]. Succeeding the dissociation of protons from the His-box and their discharge into the virus interior, the AM2 channel reverts to conformers resembling a neutral pH structure (C_closed_) for a subsequent cycle of proton shuttle [126,127,129,136].

M2-blockers are thought to block virus replication after the influenza virus infection has taken place through the prohibition of proton influx from the endosomes into the virus interior, accordingly halting virus uncoating, such that the viral genetic material cannot replicate [22,150].

### 3.3. AM2 Channel Inhibitors

Amantadine (Symmetrel) was approved by the food and drugs board (FDA) in 1966, followed by rimantadine (Flumadine) in 1994 for both treatment and prevention of IAV. They are only effective against IAV, and their utility is limited by association with severe side effects on the central nervous system (CNS) as well as the emergence of drug-resistant viruses [106]. The drug-binding site has previously been predicted by mutagenesis and electrophysiological studies, which suggested that drug-resistant mutants (V27A, A30T, S31N, and G34E) bind to the *N*-terminal pore of the TM domain [22,151].

Recently, numerous NMR and X-ray crystal structures of the intracellular TM domain have been resolved [146]. They suggest that Adamantane fits into the central cavity of the AM2 channel above the His37 box to prevent the conformational change from C_open_ to C_closed_ conformers, thus obstructing the proton conductance [128,142].

**Figure 5:**
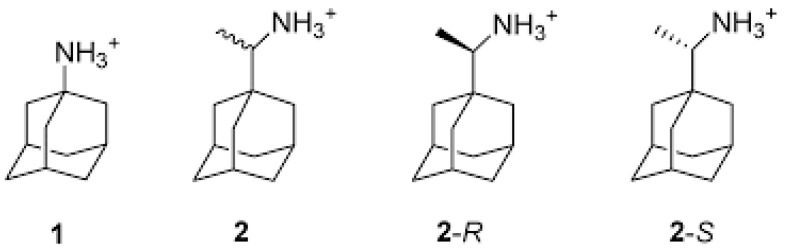
Adamantane and its amine analogues: amantadine (**1**), rimantadine (**2**), *R*-rimantadine (**2**-*R*) and *S*-rimantadine (**2**-*S*) (prepared by authors).

Amantadine and rimantadine are amphiphilic, comprised of a hydrophilic amine and a hydrophobic adamantyl or adamantylethyl cage (Figure 5). Rimantadine has a chiral center and is clinically administered as a racemate. The solution NMR spectroscopy structure of the rimantadine–AM2 channel complex indicated that inhibition occurred by an allosteric mechanism. Four rimantadines were bound to the *C*-terminal on the lipid facing surface of the helices and tightly packed to block the *C*-terminal end of the channel [120]. R-rimantadine was found to exhibit full occupation of the *C*-terminal end, thus causing higher inhibition activity of the AM2 channel than S-rimantadine [152].

The X-ray crystallographic structure of the amantadine–AM2 channel complex showed that amantadine binds to the *N*-terminal domain. The large hydrophobic group comfortably fits into the center of the aqueous cavity and physically blocks the pore, thus interrupting highly structured water networks and disturbing the protonation equilibrium of His37. This blockage suggests a physical occlusion mechanism of inhibition [121]. The amantadine cage fits into the channel pore with exceptional geometric complementarity. Amantadine fits better in the inward configuration with its amine facing towards but not directly contacting His37. pKa of His37 is affected by amantadine binding [127].

This proposed binding model (physical occlusion mechanism) is consistent with the stoichiometry of binding and the location of drug-resistant mutants (V27A, A30T, S31N, and G34E) which bind to the *N*-terminal domain, suggesting a physical occlusion mechanism for inhibition [22,153,154]. Physical occlusion is also coherent with indications that when the ammonium group of amantadine is replaced with a bulky secondary alkylamine, its effectiveness is retained [146]. The hydrophobic substituents can similarly replace the adamantine cage. Although, the positively charged primary ammonium group shows an optimal high-binding affinity when compared to tertiary amines, alcohols, and other neutral head groups, which tend to have a lower binding affinity [155,156,157].

The optimum binding affinity of primary amines suggested that the positively charged ammonium group may mimic positively charged hydronium ions produced as protons permeate through the channel to reach His37-box. The hydrated ammonium or hydronium ions are stabilized by water-mediated hydrogen-bonding [137,141,157].

The binding of amantadine to the channel causes structural and dynamical modifications to the channel by disrupting the continuous water networks that are vital for proton conductance [127,148]. The SSNMR structure of the adamantine–channel complex presented a significant decline in water–protein cross-peak by 47% compared to the open state upon drug binding, demonstrating channel dehydration, thereby preventing proton conductance. These findings indicate that amantadine binds into the pore instead of the surface, as suggested by the solution NMR study of AM2 [120,158].

These findings are in exceptional agreement with the high-resolution SSNMR structure of the amantadine–AM2 channel complex in lipid bilayers at high pH, which indicates that amantadine physically occludes the AM2 channel [122]. The crystallographic structures are also in excellent agreement with numerous functional and spectroscopic data and provide a basis for developing new anti-viral drugs against influenza viruses [22,136].

Vaccination provides the best method for the prevention and control of influenza and normally elicits a potent neutralizing antibody response [159]. The immunogenicity of M2e was first investigated in 1988 by Zebedee et al., in which to gain an understanding of the M2 protein function in the influenza virus’ replicative pathway, their study produced and characterized a monoclonal antibody to M2 [160]. This monoclonal antibody (14c2) recognized the ectodomain of the protein, and it was able to spot M2 on the virions, thus reducing viral growth through the size reduction in lytic plaques [161]. Manzoor et al. in 2020 [162] examined the anti-viral activity of monoclonal antibody rM22223 and found that rM2ss23 inhibited A/Aichi/2/1968 (H3N2) (Aichi) but not A/PR/8/1934 (H1N1) (PR8) replication. Amino acid residues at positions 54 and 57 in the M2 cytoplasmic tail were also discovered to be important for the sensitivity to rM2ss23.

Employing the amino acid sequence of the rM2ss23 variable region, Okuya et al. constructed mouse–human chimeric rMss23 (ch-rM2ss23) IgA and IgG, which were presumed to identify the same epitope, and compared their inhibitory activities in vitro [163]. The results indicated that IgA restricts virus budding more proficiently than IgG and suggested a contribution of IgA in cross-protective immunity. More so, it has been discovered that M2e-specific IgGs mouse monoclonal antibodies inhibit the plaque growth and infectivity of A/Udorn/72 in vitro [164]. Filament formation was repressed by treatment of A/Udorn/72 infected cells with M2e-specific IgG2a and IgG1 monoclonal antibodies and resulted in the fragmentation of pre-existing filaments.

Peptides have also been studied for at least 40 decades, and a broad spectrum of biological activities has been described so far. The development of antiviral peptides has been attracting much attention in recent years due to their relative safety and lower development costs in comparison with those associated with small-molecule- or antibody-based antiviral drugs [165]. The derivative (M2 MH) of M2 AH has been established to instigate viral membrane distortion and it effectively eliminated the infectivity of influenza viruses, demonstrating its potential as an antiviral peptide [166]. Membrane distortion was caused by the deep introduction of the peptide into the membrane.

## 4. Neuraminidase (NA)

The activity of NA to remove influenza virus receptors adhered to erythrocytes was discovered by Hirst [153] in studies on hemagglutination. Influenza virus receptor studies by Gottschalk [28] identified this enzyme as NA, which was eventually revealed to be involved in the spread of infection from cell to cell [167,168]. The receptor-destroying enzyme (RDE) from *Vibrio cholerae* culture fluid was found to be a source of NA [169].

Influenza virus NA (EC 3.2.1.18) catalyzes the cleavage of α-(2-3 or 2-6)-ketosidic linkage between terminal sialic acid (*N*-acetyl-neuraminic acid) and adjacent surface glycoprotein [28,168]. Cleavage facilitates the budding of the newly formed viral particles from the surface of the infected cell and prevents their aggregation on the host cell surface. The cleavage promotes the release of progeny virus to infect new host cells and spread infection in the respiratory tract mucins [154,170].

To date, 11 IAV subtypes of NA are recognized by the Centers for Diseases Control and Prevention. Of these, nine subtypes (N1–N9) are circulating in wild aquatic birds, and two more (N10 and N11) were recently found in bats [37]. N1–N9 subtypes are further divided into two phylogenic groups on the basis of their sequences and the sialic acid-binding pocket (150-loop) conformational differences. Group 1 NA *apo*-structures are in an open conformation, with a 150-cavity (residues 147 to 150) formed by the opening of the 150-loop (excluding N1 of the 2009 H1N1 pandemic), while all group 2 NA *apo*-structures lack this cavity. Group 1 comprises the N1, N4, N5, and N8 subtypes, while group 2 consists of the N2, N3, N6, N7, and N9 subtypes [171,172,173]. NA-like (N10 and N11) genes from bats are genetically distinct from NA molecules ascertained on established influenza A viruses (N1–N9), thereby creating a distinctive cluster, which is termed group 3.

### 4.1. Structure and Function of NA

High-resolution structures of NA have led to the successful design and worldwide approval of NAs. Crystal structures of all group 1 NAs (N1 [174,175], N4 [174], N5 [176], and N8 [174] and group 2 NAs (N2 [171] and N9 [177]) have been ascertained, except for N3 and N7, where attempts for crystallization have been unsuccessful. Influenza B NA crystal structures have also been established [71]. The structures of the nine N subtypes have a similar topology and share 50–70% amino acid sequence similarities [174].

**Figure 6:**
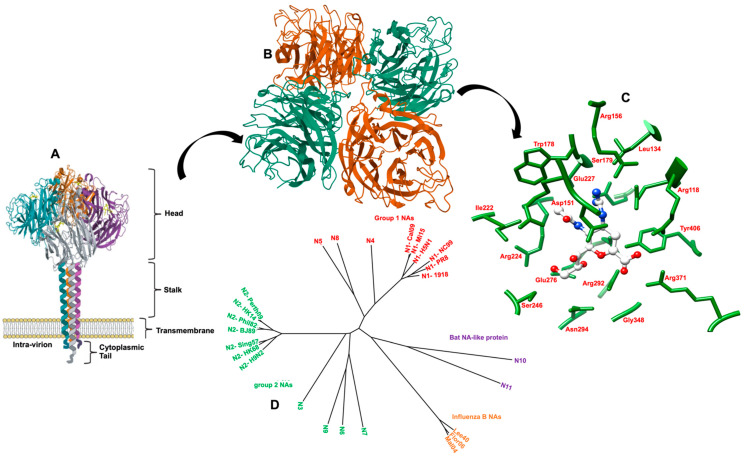
(**A**) The structure of NA as a tetramer of 4 identical monomers. Each monomer consists of 4 different structural domains called catalytic head, stalk, transmembrane and the cytoplasmic tail. The head domain structure was generated in Pymol using structural information from protein data bank code 4GZX [182]. (**B**) Top-down view of the NA tetramer. (**C**) The active site of NA in complex with Zanamivir is represented. Residues involved in catalysis are shown as green sticks (adapted with permission from ref. [183]). (**D**) Tree of known influenza virus NAs and NA-like proteins (N10 and N11). Influenza NAs cluster into group 1 (N1, N4, N5, N8) and group 2 NAs (N2, N3, N6, N7, N9). Influenza B NAs as well as the NA-like proteins (from sequences found in bats) form their own clusters (adapted with permission from ref. [184]).

NA is a type II integral membrane glycoprotein, assembling as a tetramer comprised of four identical disulfide-linked polypeptide chains. Each monomer has a molecular weight of 60 kDa and is made up of 470 amino acid residues [171,178]. NA exists as a mushroom-shaped homotetramer (240 kDa) on the virion surface, with the head atop a rod hydrophobic stalk anchoring it onto the viral surface (Figure 6). The head domain is box-shaped. Each monomer has a topologically identical six-bladed propeller-like structure. Each blade comprises four antiparallel strands of β-sheets [179,180]. The viral particle bears around 50 copies of tetramers that can form bundles on the viral surface [181].

The three-dimensional structure of NA shows that each monomer is folded into four unique structural domains. The cytoplasmic tail is critical for NA transport and incorporation into virions, while the transmembrane domain is responsible for attaching the NA to the viral envelope. The stalk domain is accountable for connecting the head to the transmembrane domain. Lastly, the catalytic head ectodomain attached to the C-terminus of the stalk carries the enzyme active site for sialic acid cleavage and other essential antigenic amino acids.

The structure of the tetrameric head domain has been determined for all nine NA subtypes by X-ray crystallography. The active site forms a shallow cavity at the surface and center of each monomer. It is positioned in a sideways conformation, which allows it to cleave sialic acids from adjacent membrane glycoproteins. This sialic acid binding site is well-formed, large and rigid, with an unusually large number of charged amino acid residues which cluster in the cavity and around its rim [34].

The inner cavity is comprised of eight highly conserved catalytic residues that interact directly with sialic acids responsible for the catalytic activity of the enzyme (Arg118, Asp151, Arg152, Arg224, Glu276, Arg292, Arg371, and Tyr406) (N2 numbering) [170,181]. Additionally, the rim consists of 10 highly conserved structural residues (Glu119, Arg156, Trp178, Ser179, Asp (or Asn in N7 and N9) 198, Ile222, Glu227, Glu277, Asp293, and Glu425) responsible for the stabilization of the structure. Two calcium-binding sites located near the active site are responsible for the stabilization of the tetramer at low pH conditions [185,186,187].

**Figure 7:**
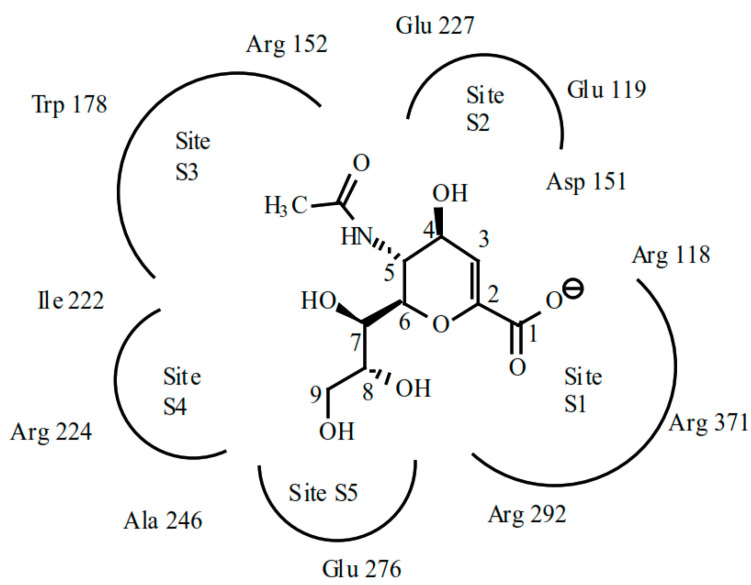
Diagram of NA active site with dehydrodeoxy-*N*-acetylneuraminic acid (DANA) inhibitor showing the five inhibitor binding sub-sites and nearby critical residues [188].

The eight conserved catalytic residues are organized in a sequence of interlinked pockets that determine the mode in which the enzyme interacts with sialic acid. The active site is divided into five regions, termed subsites (S1 to S5), derived from the crystal structure of the substrate-based inhibitor DANA (dehydrodeoxy-*N*-acetylneuraminic acid) bound to the active site (Figure 7). Subsites S1, S2, S3, and S5 are occupied, while any portion of the DANA-based inhibitors does not occupy subsite S4.

Site S1 comprises a cluster of positively charged arginine triad residues: Arg118, Arg292, and Arg371. Site S2 forms a negatively charged region derived from Glu119 and Glu227 residues. Site S3 is a small hydrophobic region derived from Trp178 and Ile222 sidechains and a hydrophilic region provided by the Arg152 sidechain and a bound water molecule. Site S4 is primarily a hydrophobic region formed from the sidechains of Ile222, Ala246, and the hydrophobic face of Arg224. Site S5 creates an area of mixed polarity, derived from Glu276 and Ala246 residues.

### 4.2. Catalytic Mechanism of NA

**Figure 8:**
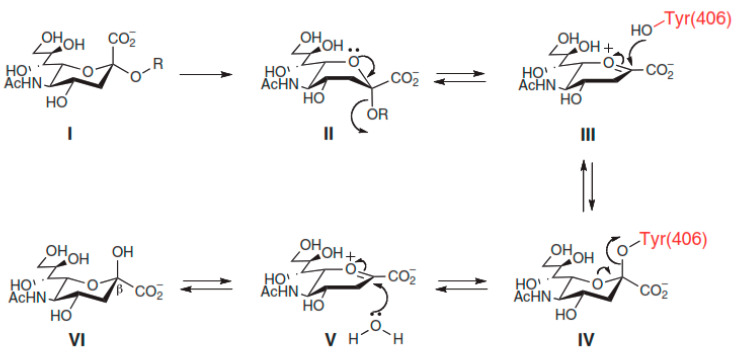
Catalytic Mechanism of NA showing four major steps of NA Inhibitors [190].

The catalytic mechanism of NA has been studied in some detail but is still not completely elucidated. Still, based on structural information and biochemical studies, it has been suggested that the catalytic mechanism for the cleavage of sialic acid from glycoconjugate has four major steps (Figure 8). The first step is the binding incidence. The substrate binds to the active site, resulting in salt-bridge formation between the carboxylate of the substrate and the triarginyl cluster of the active site. The binding of sialic acid to the catalytic site distorts the pyranose ring from the preferred chair conformation to a pseudoboat conformation. This is a result of a strong ionic, hydrogen bond and steric interactions [34,189]. The carboxylate group of the bound sialic acid adjusts from the axial into the pseudoequatorial position due to strong ionic interactions with the three arginine residues, 118, 292, and 371, resulting in the formation of a sialosyl cation (oxocarbenium ion) at the C2 atom of sialic acid.

The second step of the catalytic reaction is the formation of the endocyclic sialosyl cation transition state intermediate. It requires proton donation from solvent aided by negatively charged amino acid residues. It is believed that the hydrogen-bonding network of water molecules and protein residues leading from a charged group on the protein surface to water molecules could facilitate proton donation [191]. Asp151, Arg152, and Glu277 residues are thought to stabilize the positive charge of the sialosyl cation. Covalent interaction of the sialosyl cation with the hydroxyl group of Tyr406 at the base of catalytic site is also believed to contribute to the stability of the cationic intermediate.

The final two steps of the enzymatic mechanism encompass the formation and release of sialic acid. Stable sialosyl cation intermediate favors the cleavage of the glycosidic bond, yielding sialic acid and the aglycon molecule. The release of sialic acid from the active site is favored by the mutarotation of β-anomer conformation to a thermodynamically more stable α-anomer conformation for sialic acid in solution. The aglycon molecule leaves the enzyme active site with the glycosidic oxygen [192,193].

The optimum NA activity was observed to occur at a pH range of 5.5–6.6 and a temperature of 37 °C. It has also been suggested that the presence of calcium ions adjacent to the active site is essential for both the activation and thermostability of NA [185]. Moreover, the existence of highly conserved amino acid residues in the active site makes it an attractive target for drug design as it accords the development of transition-state analogues that inhibit NA.

**Figure 9:**
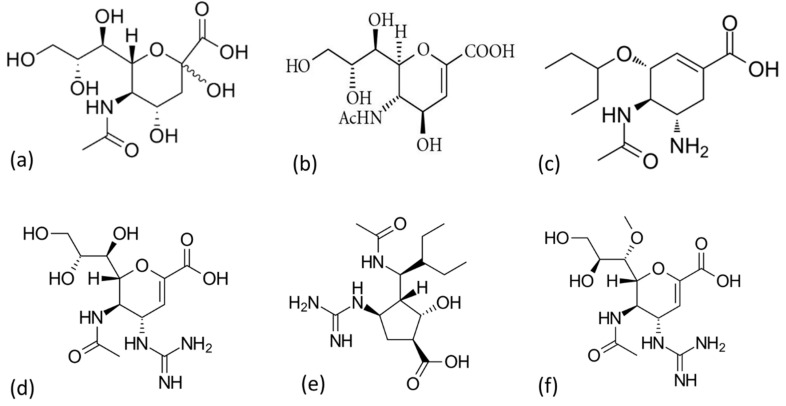
Chemical structure of sialic acid for treatment and prophylaxis of influenza infection (**a**), DANA (**b**), zanamivir (**c**), oseltamivir (**d**), peramivir (**e**), and laninamivir (**f**) (prepared by authors).

NA inhibitors are effective against both IAV and IBV. They prevent NA from cleaving the sialic acid, thus budding viral particles remain attached to the surface of the infected cell and each other. This results in the suppression of infection to one round of replication [168]. There are three classes of globally approved NA inhibitors (zanamivir, oseltamivir, and peramivir) for the treatment and prophylaxis of influenza infection (Figure 9). Their design is based on the transition state analogue of sialic acid (2-deoxy-2,3-dihydro-*N*-acetylneuraminic acid or DANA) developed in the 1970s, which displayed low binding affinity into the active site [179,194].

This critical finding paved the way for the design and development of drugs that closely mimic DANA and fit in the active site pocket to hinder NA activity. Further advances in technology and different techniques led to the discovery of high-resolution crystal structures of both NA and sialic acid [178,195]. Protein X-ray crystallographic study of the complex NA with DANA has aided the identification and characterization of the site of enzyme catalysis. It displayed the presence of an empty positively charged cavity in the active site, which aligned with C4 of the bound sialic acid. The findings led to suggestions that the introduction of a positively charged group to the C4 of DANA might enhance binding affinity to the catalytic site [170,196,197,198].

Zanamivir (Relenza) was the first potent NA inhibitor to be approved by the FDA in 1999. DANA-based zanamivir synthesis involved the substitution of C4-OH with the 4-guanidino group, which showed a 1000-fold better binding affinity into the active site pocket over DANA. Zanamivir (4-guanidino-DANA) is administered via oral inhalation directly into the respiratory tract. However, it has low bioavailability due to the presence of the guanidino group [197].

The FDA subsequently approved oseltamivir (Tamiflu) in the year 1999 to address the low bioavailability limitation of zanamivir. The development of orally bioavailable oseltamivir involved two substitution mechanisms of the DANA cyclohexene ring: the substitution of the C6 glycerol sidechain of DANA with a bulky hydrophobic pentyl ether sidechain, and the C4-OH substitution with an amino group rather than guanidino. Oseltamivir is orally administered as a prodrug of oseltamivir phosphate and converted to an active metabolite, oseltamivir carboxylic acid, by endogenous esterase [199,200,201].

The FDA globally approved Peramivir (Rapivab/Rapiacta/Peramiflu) in the year 2010. Peramivir is also derived from DANA and contains a cyclopentane ring with features of both zanamivir and oseltamivir, the C4-guanidino group, and the bulky hydrophobic pentyl ether sidechain, respectively. Such features lead to multiple interactions (higher binding affinity) with the NA catalytic site. This drug is administered intravenously due to low oral bioavailability [202,203,204,205].

Laninamivir (Inavir) is currently licensed for use in Japan since the year 2014 and is undergoing Phase III clinical trials in other countries. It is a derivative of zanamivir, and it contains the C4-guanidino group and an additional 7-methoxy group. Laninamivir is administered as a prodrug (laninamivir octanoate) via nasal inhalation and converted to an active metabolite (laninamivir) by endogenous esterase. It has long-lasting anti-viral inhibition with activity against oseltamivir-resistant viruses [206,207,208].

With regards to new antivirals for targeting NA, acylhydrazone has been considered a fortunate structure capable of offering ligand points for more than one type of bio-receptor. Zhao et al. [209] discovered that some acylhydrazone derivatives exhibit better inhibition than oseltamivir carboxylate against NA. Furthermore, Yu et al. [210] also designed and synthesized benzoylhydrazone NA inhibitors with higher NA inhibitory activity to the positive control oseltamivir carboxylic acid. Li et al. [211] likewise designed and synthesized novel acylhydrazone NA inhibitors, with most of them exhibiting good inhibition activity with a significantly lower activity than that of the positive control oseltamivir carboxylic acid.

Modifications of oseltamivir that enable higher affinity binding at the amino acids forming the 150- or 430-cavity could yield novel NA inhibitors that are not sensitive to common mutations of NA [212]. Moreover, Ju et al. [213] designed and synthesized 27 oseltamivir analogues by modification at the C-1 position to research the chemical space around the 430-cavity. Compound 8b indicted the best inhibitory activity against H5N1 and H5N6 NAs. Xie et al. [214] also discovered group-1-specific NA inhibitors that are involved in fighting the H5N1 virus. Derivatives of oseltamivir were designed and synthesized by targeting the 150-cavity. Among the synthesized derivatives, compound 20l showed higher inhibitory efficacy against NAs from three H5N1 viruses. The inhibitory activity was better than that of oseltamivir carboxylate.

In 2019, Ji et al. [212] designed and synthesized oseltamivir derivatives by exploiting the 150-cavity in NAs. The compounds exhibited antiviral activities with higher potency (5- to 85-fold) than those of oseltamivir carboxylate against N1, N8, and N1-H274Y mutations. Jia and colleagues [212] also explored the chemical space of both 150-cavities in NAs, and oseltamivir derivatives were designed, synthesized and evaluated by modifying the C1 and C5 amino group of oseltamivir carboxylate. The most effective N1-selective inhibitor exhibited 1.5- and 1.8-times greater activity than oseltamivir carboxylate against H5N1 and H5N1-H274Y.

## 5. Conclusions and Future Perspectives

The transmission of human influenza through inter-continental circulation makes surveillance a vital member in the global management of influenza. The broad host range of influenza virus and interspecies transmission are essential factors for its continual spread and genetic variation. The transitional reservoirs such as pigs, birds, ducks and horses play a critical function in keeping the influenza virus in nature and facilitating its transmission to humans. Thus, other than constant surveillance and developing a universal vaccine and potent antivirals, prolific global management of such reservoirs to limit the circulation and formation of new infectious influenza virus variants is necessary.

Although a considerable amount of biochemical and lower-resolution structural information has been attained for the AM2 proton channel, many rhetorical questions persist about this versatile protein. The present high-resolution structure offers a foundation for elucidating the mechanism of proton conduction through the AM2 channel. However, advancement in the discovery of new inhibitors targeting mutants of the AM2 channels has been sluggish. Recent developments in understating the structure and vital properties of the AM2 channel in a lipid bilayer, as well as the interaction of amantadine with the channel, have stimulated structure-based drug design and computer-aided drug design.

The resistance to NA inhibitors by influenza viruses is an emerging problem of high epidemiological and clinical impact. The emergence of drug resistance to inhibitors of NA, such as oseltamivir and zanamivir, qualifies a necessity for an alternative strategy. The alternative strategies are predominantly essential to recognize viable NA inhibitors which may not only have improved antiviral activity, but can also endure the threat of resistance.

There is advancement in the development of new NA inhibitors, but there has been slow progress with AM2 proton channel inhibitors. Modifications of the subunits bonded to its acyl and imine functions of acylhydrazone result in several derivatives, which confers diversity of molecular targets and provides more therapeutic properties. The 430-cavity widely exists in a variety of subtypes, including group-1 and group-2, and could provide greater chemical space for further modification. Approaches to broaden the availability of novel antiviral compounds include the development of synthetic peptides that disrupt the entry of viruses into cells. Although antibodies specific for M2 are unable to bind efficiently to free virus particles and thus do not neutralize virus infectivity, they can bind to M2e expressed on the surface of virus-infected cells and thus are a potential antiviral tool for preventing new virion release. Further studies on humans are needed to understand the protective role played by anti-influenza protein antibodies during infection or vaccination. That information will greatly enhance our understanding of how current influenza vaccines could be improved to provide cross-protective immunity in humans.
